# Exploring primary care doctors’ perceptions on their roles in palliative care in Singapore: a qualitative study

**DOI:** 10.1186/s12875-026-03330-5

**Published:** 2026-04-21

**Authors:** Yae Sol Park, Ariffin Kawaja, Wei Teen Wong, Victoria Hwei May Wong, Chirk Jenn Ng

**Affiliations:** 1 SingHealth Polyclinics, Singapore, Singapore; 2 SingHealth-Duke NUS Family Medicine Academic Clinical Programme, Singapore, Singapore; 3 National Cancer Centre Singapore, Singapore, Singapore; 4 Lee Kong Chian School of Medicine, Singapore, Singapore

**Keywords:** Primary care physicians, Palliative care, End-of-life care, Perceptions

## Abstract

**Background:**

Singapore faces a rapidly aging population with increasing multimorbidity, creating an urgent need for expanded palliative care services. Despite primary care physicians (PCPs) being ideally positioned to provide palliative care throughout patients’ disease trajectories, their role remains underdeveloped in Singapore’s healthcare system. This study aimed to explore PCPs’ perceptions of their roles in providing palliative care to patients with life-limiting illnesses in Singapore’s primary care setting.

**Methods:**

We conducted a qualitative study at two SingHealth Polyclinics with distinct patient population demographic profiles. Using purposive sampling, we recruited 20 PCPs with at least 12 months of primary care experience, representing various professional designations. Data were collected through in-depth interviews and focus group discussions from September to December 2023. All interviews were audio-recorded, transcribed verbatim, and analysed using a thematic approach.

**Results:**

Our analysis revealed a dichotomy between PCPs’ recognition of their potential roles in palliative care and their reservations about fulfilling these responsibilities. While participants identified several important functions they could serve—including needs assessments, symptom management, advance care planning, psychosocial support, and care coordination—many expressed significant concerns about implementation. These perceptions were shaped by uncertainty regarding their place in the palliative care ecosystem, concerns about patient and specialist expectations, and personal comfort levels with end-of-life discussions. Additionally, some PCPs perceived that patients viewed them primarily as providers for routine or chronic care, not for managing complex end-of-life needs, which further affected their sense of role clarity in palliative care.

**Conclusions:**

PCPs in Singapore recognise their potential contributions to palliative care but experience uncertainty regarding their roles in current primary care settings. These perceptions are shaped by variability in training and experience, unclear role boundaries, and patient and specialist expectations. Strengthening role clarity, training, and collaboration with specialist services may support the integration of palliative care into primary care.

**Supplementary Information:**

The online version contains supplementary material available at 10.1186/s12875-026-03330-5.

## Introduction

The global burden of serious health-related suffering is projected to increase significantly—from 26 million people in 2016 to 48 million by 2060—an 87% rise largely affecting adults aged 70 and older [1]. This increase reflects the aging global population and the rising prevalence of non-communicable diseases, leading to a greater number of individuals living with life-limiting illnesses [2]. These conditions, which include cancer, chronic organ failure, dementia, and frailty, are defined as illnesses for which there is no reasonable hope of cure [3, 4].

Singapore is experiencing a rapid demographic shift, with nearly one in four residents expected to be aged 65 or older by 2030 [5]. Multimorbidity among older adults is also rising, with the proportion of those aged ≥ 60 living with three or more chronic conditions increasing from 19.8% in 2009 to 37% in 2017 [6]. These trends underscore a growing need for comprehensive and accessible palliative care services.

Despite expansion in Singapore’s palliative care sector, major gaps in timely access remain, particularly for patients with non-cancer life-limiting illnesses [7]. With Healthier SG - Singapore’s healthcare reform initiative launched in 2023 that shifts the delivery strategy from curative to preventive care and strengthens primary care as the foundation of healthcare delivery - the approach to healthcare has evolved, placing renewed focus on primary care and community care, and redefining roles and priorities within the healthcare system [7]. A key challenge is delayed referrals to palliative care services, including hospice care, with the average time between referral and death being only 22 days [8]. This is shorter for non-cancer patients (11–13 days) compared to cancer patients (27–35 days) [8].

Globally, palliative care remains unevenly implemented. In 2017, only 14% of the world’s population had access to advanced palliative care services, mostly in high-income countries [9]. A global mapping study of 198 countries found that only 30—mainly from Western Europe, North America, Australia, and New Zealand—had achieved advanced integration of palliative care into their health systems. The rest had limited or no provision, due to variable national commitment, resource allocation, and system-level integration [9]. In Europe, barriers to implementing palliative care in the community include limited GP training, inadequate reimbursement models, prescribing restrictions, insufficient specialist support, and poor identification of patients needing care [10].

Primary care is increasingly recognized as key to delivering effective palliative care. Positioned at the frontline, primary care physicians (PCPs) manage chronic diseases across their trajectories and the life-course of patients, and often have established, trust-based relationships with patients [11]. The WHO framework on integrated, people-centred health services positions palliative care as a core component of primary care [12]. PCPs are well-suited to provide holistic, continuous care, aligning with the principles of both primary and palliative care [13, 14], and serve a vital role in care coordination across settings [15].

Patients with life-limiting illnesses often value continuity, accessibility, and established relationships with their PCPs, particularly as their condition progresses [11, 12]. They may expect PCPs to play a role in symptom management, advance care planning, and coordination of care across settings, while maintaining holistic and person-centred care [11–15]. In addition, patients and caregivers value clear communication, emotional support, and guidance in navigating complex healthcare decisions towards the end of life [16–18].

In Singapore, structured palliative care training is available for PCPs and allied health professionals through institutions such as the Lien Centre for Palliative Care, the Palliative Care Centre for Excellence in Research and Education, and NUS Division of Graduate Medical Studies [19–21]. Building on these efforts, the Ministry of Health’s 2023 National Strategy for Palliative Care outlines a vision to expand community-based palliative services and better integrate palliative care into primary care settings [7].

However, successful implementation requires understanding PCPs’ perceptions, as their engagement is crucial for effective delivery. Exploring how PCPs view their roles in palliative care will help inform targeted implementation strategies and policies. This study, therefore, aimed to explore PCPs’ perceptions of their roles in delivering palliative care to patients with life-limiting illnesses in Singapore, and to identify opportunities to enhance their involvement in meeting the needs of an aging population.

## Methods

### Study design

This study employed a qualitative methodology to explore PCPs’ perceptions of their roles in palliative care within Singapore’s public primary care settings. Given that integrating palliative care into primary care remains an emerging concept in the local context, an inductive qualitative approach was chosen to allow for a rich, nuanced understanding of doctors’ roles from their perspectives. Data collection combined individual in-depth interviews and focus group discussions to capture both individual experiences and collective insights. This approach enabled exploration of how PCPs conceptualize their roles in palliative care delivery, their current involvement with patients having life-limiting illnesses, and their vision for potential expanded roles in Singapore’s palliative care ecosystem.

### Study sites

This study was conducted at two SingHealth Polyclinics, a public primary care institution comprising nine polyclinics that collectively serve over 1.3 million residents in Singapore. The selected sites—Outram and Sengkang Polyclinics—have distinct profiles: Outram, located in central Singapore, serves an older urban population, while Sengkang, in the northeast, caters to a younger demographic, including more families and working adults. The contrasting patient demographics across these two clinics are likely to influence providers’ exposure to and experience with palliative care. These polyclinics also differ in manpower, infrastructure, and resources, with Sengkang seeing approximately 900–1000 patients daily. This diversity would enable the research team to contextualize findings across varied care settings.

### Study population

The study population comprised doctors providing primary care services in SingHealth Polyclinics for at least 12 months, including both part-time and full-time practitioners. Eligible participants held positions as resident physicians, family physicians, locums, associate consultants, consultants, or senior consultants. Relief doctors, nurses, pharmacists, and allied health workers were excluded from this study. This population was selected to ensure participants had sufficient experience within the polyclinic system while representing the full spectrum of physician roles in this primary care setting.

### Recruitment

Lists of eligible doctors were obtained from clinic heads and executives following site initiation visits, as approved by the Institutional Review Board. Participants were invited via email to join either in-depth interviews (IDIs) or focus group discussions (FGDs), with assurance that participation was voluntary with no impact on their work. Interested doctors received information sheets and consent forms electronically, with at least one week provided for consideration before providing written informed consent and completing socio-demographic data forms. Recruitment and interviews occurred from September to December 2023. We employed purposive sampling across two polyclinics, selecting participants based on years of primary care experience, postgraduate training, and professional designation to capture diverse perspectives on palliative care delivery. Recruitment continued until no new themes appeared to emerge from the field notes and in the ongoing analysis.

### Interview guide

The interview guide was developed based on the Integrated Behavioural Model (IBM) [22], a literature review, and discussions among the study team. It explored PCPs’ views and experiences in providing palliative care to patients with life-limiting illnesses, aiming to identify barriers and facilitators in routine practice. The guide was flexible, allowing interviewers to ask follow-up questions, probe for details, and seek clarification. Questions were refined iteratively as interviews progressed to capture emerging themes. The guide comprised 18 main questions across seven domains, with additional probes to elicit further details. The full interview guide is provided in Supplementary File 1 – Interview Guide for Primary Care Physicians.

### Interviews and transcribing

An experienced qualitative researcher (NCJ) conducted the first interview with observation by the principal investigator (PYS), a Family Medicine fellowship trainee with prior qualitative research training. PYS conducted subsequent interviews under the direct supervision of NCJ and AK, who provided feedback through direct observation. Interviews were conducted individually in private rooms at the polyclinics to encourage frank disclosure. The semi-structured interview guide explored PCPs’ views and experiences of providing palliative care to patients with life-limiting illnesses in the primary care setting. Before each interview, PYS briefed participants on voluntary participation, confidentiality, and the importance of honest opinions. Participants were referred to by study identification numbers to maintain anonymity. Throughout the sessions, PYS maintained a neutral stance while practicing empathetic listening and attending to both verbal responses and non-verbal cues. Participants were given sufficient time to freely express their perspectives without interference.

All interviews were audio-recorded and transcribed verbatim by a professional transcriber. In addition to the recordings, PYS took interview notes to document key observations and emerging themes. PYS subsequently audited each transcript against the original recordings, corrected any errors, and prepared the edited transcripts for analysis. Participants received grocery vouchers worth SGD20 (approximately USD15) as compensation for their time.

### Data coding and analysis

Data collection and analysis were conducted concurrently using an inductive thematic approach, guided by the research questions. Three study team members (PYS, NCJ, AK) independently analysed the first two transcripts to develop the coding framework. Subsequent analysis of the remaining transcripts was done by one researcher (PYS) using the coding framework; any proposed new themes or changes in the coding framework were discussed at the regular research meetings before any changes were collectively agreed upon. During the analysis, the researcher read and re-read each transcript to familiarise themselves with the content. Open and axial coding was done; the researcher analysed the transcripts line by line and sentence by sentence, labelling segments with ‘open codes’ while keeping the research questions in mind. Once a list of codes was generated, the researchers went through the codes and grouped those with similar concepts together to form categories (axial coding) [23]. A coding framework was formed subsequently after discussion among the study team members. Constant comparisons were made across different transcripts to ensure the accuracy of the analysis [24]. Categories and themes were identified from the analysis to answer the research questions. Complementing the interview data, field notes were taken by the researchers during and after each interview or focus group discussion to capture new emerging issues and personal reflections. These field notes served as supplementary data for triangulation with the interview findings.

## Results

We recruited 28 PCPs, of whom 20 agreed to participate in the study. We conducted five focus group discussions (FGDs) and one in-depth interview (IDI) involving 20 PCPs. The single IDI was conducted because one participant was unable to join the previously scheduled FGDs. Participants’ ages ranged from 30 to 54, and their years of practice in primary care ranged from 4 to 12.5 years. The duration of the FGDs and IDI sessions ranged from 21 to 63 min. The characteristics of the participants are described in Table 1.

**Table 1:** Characteristics of participants (*n* = 20)

Characteristics	*n*	%	Range
Age (years)	—	—	30–54
Years of practice in primary care	—	—	4–12.5
Gender			
Male	2	10.0	—
Female	18	90.0	—
Ethnicity			
Chinese	17	85.0	—
Indian	2	10.0	—
Other	1	5.0	—
Postgraduate training			
Graduate Diploma in Family Medicine	4	20.0	—
Master of Medicine (Family Medicine)	11	55.0	—
Fellow, College of Family Physicians (Singapore)	5	25.0	—
Designation			
Resident Physicianᵃ	2	10.0	—
Family Physicianᵇ	8	40.0	—
Associate Consultant	6	30.0	—
Consultant	2	10.0	—
Senior Consultant	2	10.0	—

ᵃ Qualified physicians with at least an undergraduate medical degree, granted full medical registration, and with at least 3 years of clinical experience

ᵇ Registered medical practitioners with relevant and recognized postgraduate qualifications (Graduate Diploma in Family Medicine, or Master of Medicine in Family Medicine) and with at least 3 years of clinical experience

The study results are organized into the following categories: palliative care exposure and experience, views and perceptions of primary care in palliative care, and roles of PCPs in palliative care. Each theme and subthemes are supported by corresponding representative verbatim quotes from the participants. A summary of the themes and subthemes is described in Table 2.

**Table 2:** Themes and subthemes

Theme	Subthemes
1. Palliative care exposure and experience	—
2. Views and perceptions of palliative care in primary care	• Perceived limited role of polyclinics in palliative care • Patient’s perception of PCPs in delivering palliative care • Perceived lack of understanding among specialists regarding challenges in primary care • Negative view of PCPs in discussing life and death with patients
3. Roles of PCPs in palliative care	• Conducting palliative care needs assessments • Managing symptoms • Facilitating Advance Care Planning (ACP) and End-of-Life (EOL) discussions • Providing psychosocial support • Ensuring smooth transitions between care settings and coordinating care

### Palliative care exposure and experience

Among the 20 PCPs interviewed, 13 expressed prior experience in managing patients with life-limiting illnesses, while the rest only had exposure during medical school postings or residency training.

#### Palliative care exposure

PCPs encountered patients at different stages of their life-limiting illness with varying palliative care needs. Although current medical training offers some exposure to palliative medicine, it tends to be more observational than direct involvement in caring for patients with palliative care needs.

#### Palliative care experience

##### Terminal cancer

PCPs have cared for patients who were at different stages of their malignancies with different treatment goals ranging from curative intent to conservative management. Patients receiving conservative care included those who declined cancer therapy at diagnosis, as well as those who had exhausted multiple lines of treatment and were no longer on active cancer therapy.

*“*I think recently I saw one patient during the general clinic, there’s this gentleman who is in stage four metastatic liver cancer. I think mets (metastasized) to the peritoneum, every part of the body already. And then he’s here today because he has a bit of constipation.*”*

(PC 16, Family Physician)

*“It was a discharge from the hospital……noted there were lung lesions*,* other lesions in the liver. Because of the patient’s age and frailty*,* they discussed with the family and decided to just conservatively manage and discharge him back to primary care.*

(PC14, Resident Physician)

##### End-stage organ failure

PCPs had encountered patients with organ failure, particularly end-stage renal failure (ESRF), who wanted to opt out of dialysis. For instance, they encountered complications due to electrolyte imbalances such as recurrent hyperkalaemia, which they struggled to manage, especially when the patient declined to be referred to the renal specialists in the tertiary hospital for management.

“A few weeks ago, a patient who doesn’t want to have dialysis for ESRF and his potassium was really high. And he doesn’t want to go to A&E”

(PC1, Senior Consultant)

“We do have those CKD patients. Maybe stage four, stage five. Sometimes they refuse to see the renal. So I mean, the issue is sometimes with the electrolytes. Usually it’s potassium that’s giving the problem. So, I mean, of course, we will try to advise them to see. But if they are not going, then we just need to control the episodes of hyperkalaemia.”

(PC10, Senior Family Physician)

### Views and perceptions of palliative care in primary care

#### Limited role of polyclinics in palliative care

Some PCPs perceived polyclinics as having a limited role in palliative care, as they believed specialized palliative care teams or home hospices should handle issues such as pain management and end-of-life discussions.

*“I never had to manage a palliative issue before*,* like pain management or discussion on end-of-life. Usually*,* that has already been sorted out by the Pal (Palliative Care) team or a home hospice. I feel that palliative care in Singapore is already quite well-established. I think the referrals from hospitals to like home hospice are very fast. So*,* therefore*,* I feel like there isn’t much of a gap. Right now*,* maybe not so much of a role that I see like polyclinic doctors playing.*

(PC5, Family Physician)

*“I always have the impression that if you are really on pal(palliative care) then maybe a home palliative*,* home hospice may be a more suitable option. So*,* I’m not sure what benefit can a polyclinic do….We are not even sure what we can do for the patient.”*

(PC2, Associate Consultant)

#### Patient’s perception of PCPs in delivering palliative care

Patients might perceive PCPs as primarily managing common ailments rather than complex issues associated with end-of-life care.

“We do need the patient to accept it (delivering palliative care in primary care) as well. Because generally think their understanding of family medicine is general and GP’s in general may not reach to the level of managing severe issues, such as, end of life care. They may still think of us as a diabetes, hypertension doctor or cough and cold doctors.”

(PC20, Family Physician)

#### Lack of understanding among specialists regarding challenges in primary care

Specialists might not fully grasp the challenges encountered in primary care, as expressed by a PCP who wanted reciprocal support from specialists to align with the increased responsibilities placed on PCPs.

“You keep getting us to do more. It’s important but also, I think our specialist friends may not understand what kind of situation we are very much in, in primary care. If they want us to do more, I think on their end they also need to do more in terms of supporting what the primary care side can do.”

(PC9, Consultant)

“Basically, they are trying to transfer their hospital problem into our problem so that it’s not their problem but it’s still a problem.”

(PC11, Senior Consultant)

#### Negative view of PCPs in discussing life and death with patients

Some PCPs were uncomfortable discussing life and death issues with patients; some viewed them as unnecessary.

“Partially like a personal conviction. Maybe to some (PCPs), it can become a professional conviction if there is official training. But I mean if like, as an individual, we are more concerned with things like existence and like the meaning of life. Personally these kinds of questions to ask are lame. Why would I want to think about death? Like why must I think so much. Then, of course we also won’t ask our patients too much about these kinds of things, I suppose.”

(PC8 Family Physician)

### Potential roles of PCPs in palliative care

The participants expressed several potential roles in delivering palliative care within their practices, including conducting palliative care needs assessments, managing symptoms, facilitating advance care planning (ACP) and end-of-life (EOL) discussions, providing psychosocial support, ensuring smooth transitions between care settings, and coordinating care. 

#### Conducting palliative care needs assessments

The PCPs felt that, as they were often the first point of contact for patients with acute issues or managing chronic illnesses, they could play a vital role in identifying patients who might benefit from palliative care during their disease trajectory.

“I think the ESRF patients who are elderly or prefer not to have any intervention, like dialysis. I think they may become symptomatic towards the end stage. They might benefit from palliative care.”

(PC14, Resident Physician)

“That patient had advanced liver cancer and was seeing us for his chronic conditions. And then, when I reviewed his records, he was only on follow-up with the surgeon. So, I think he wasn’t keen on further treatment, but I did highlight to him that he is having pain related to his cancer and that maybe a review by the palliative care physician may actually be beneficial to him.

(PC18, Associate Consultant)

#### Managing symptoms

Some PCPs expressed that they could conduct symptom reviews for various common symptoms seen in patients with life-limiting illnesses, including pain, dyspnoea, and constipation. Additionally, they could manage chronic diseases and adjust the medications as part of palliative care.

“The patient was here for other conditions. Other issues, but recognizing that she is also a patient with some end-stage tumour who may require some pain management. So, I actually enquire a bit more about her pain status or whether the pain medicine given by the hospital team is adequate to help with her pain.”

(PC19, Associate Consultant)

*“Basically*,* turn on a fan to blow air into your face*,* that can help to relieve the dyspnoea for (lung cancer patient)….It was about de-escalating his diabetes medicines with the loss of his appetite with his cancer.*

(PC20, Family Physician)

#### Facilitating ACP and EOL discussions

PCPs felt that they could facilitate discussions with patients and their families to explore goals of care, preferences for treatment, and end-of-life wishes.

*“I’ll probably ask the family whether any ACP or any care plans have been done. And then*,* I explore patients’ wishes and all that.*

(PC17, Family Physician)

*“We probably want to engage them in discussion about advance care planning*,* especially if their mental capacity is still intact. Get into details*,* like in-depth discussion with them*,* what do they want if their conditions fail or if he develops certain complications from the end-organ diseases.*

(PC20, Family Physician)

#### Providing psychosocial support

PCPs expressed that they could provide psychosocial support to patients as they navigated the challenges associated with life-limiting illnesses. They highlighted that their rapport and established relationship with patients positioned them well to offer this support.

“I think our relationship with patients makes a huge difference. Relationships that are much deeper, more real to the patient, much more supportive, spiritually supportive. I feel that actually the primary care physician who knows the patient very well, you become a source of spiritual and moral support to the patient already.”

(PC9, Consultant)

*“It’s also the comms part*,* communication*,* communication*,* communication. Because at a point where you don’t have a fancy gadget or medication to treat their illness. It’s all about talking and managing expectations and really building that trust*,* you know*,* that you’re there for them.*

(PC6, Associate Consultant)

#### Ensuring smooth transitions between care settings and coordinating care

PCPs mentioned their role in ensuring smooth transitions between different care settings and facilitating access to suitable services, such as home-based palliative care. They could also play a role in referring patients to palliative care physicians and coordinating their overall care.

“He had an undiagnosed malignancy in the peritoneal abdomen…. He declined a biopsy, then he came to the polyclinic for abdominal pain. At that time, he had some CKD stage 3…So in view of his symptoms and possibly requiring …opioids in the future, he was referred to home hospice…. I did a back-end referral to home hospice.”

*(PC7*,* Family Physician)*

“We can also direct them to the palliative team. Or to some resources like Singapore Cancer Society. They may have some educational resources, they may have talks, they may have like support groups, caregiver training, etc.”

*(PC20*,* Family Physician)*

## Discussion

Our findings reveal a dichotomy between PCPs’ recognition of their potential roles in palliative care and their reservations about fulfilling these responsibilities. While PCPs identified important functions they could serve—including needs assessments, symptom management, advance care planning, psychosocial support, and care coordination—they expressed significant concerns about assuming these duties.

Several factors shape these perceptions. The belief that specialized palliative care teams should manage EOL care reveals uncertainty among PCPs regarding their place in the palliative care continuum [25]. This uncertainty mirrors findings from other healthcare systems where role ambiguity has been identified as a significant barrier to primary care involvement in palliative care, particularly among providers caring for non-cancer patients [26]. Cultural perceptions further complicate this uncertainty. Patients’ views of PCPs’ ability to deliver palliative care in Singapore may be influenced by the generalist role traditionally associated with primary care. This contrasts with findings from other healthcare systems where patients more readily accept PCPs’ involvement in EOL care [26]. To address these perceptions, healthcare systems require structured approaches, including patient education about the comprehensive scope of primary care services and visible integration of PCPs in palliative care teams. International evidence suggests that implementing shared care models where PCPs work collaboratively with specialists can effectively establish PCP credibility in palliative care delivery [27].

Communication gaps between care settings exacerbate role perceptions, with PCPs expressing frustration about specialists’ limited understanding of primary care constraints [28]. The sentiment that specialists are “trying to transfer their hospital problem into our problem” indicates tensions requiring improved inter-professional collaboration [29]. These communication challenges can be addressed through structured interventions that promote mutual understanding and clearer role definitions [30]. For example, establishing clear protocols for specialist-primary care involvement in palliative care planning, creating standardized handover procedures that specify ongoing responsibilities, and implementing regular inter-professional meetings have been identified as key strategies to improve specialist-generalist collaboration [7, 29].

Personal discomfort with mortality discussions emerged as another significant barrier. This aligns with research showing that healthcare providers often avoid EOL discussions due to personal discomfort or insufficient training [31], despite evidence that these conversations lead to less aggressive medical care near death and better bereavement outcomes for caregivers [16]. Structured communication training programs can systematically address this barrier, with simulation-based approaches showing particular effectiveness in primary care settings [17].

Despite these reservations, PCPs recognize their established relationships with patients and families uniquely position them to provide psychosocial support and maintain continuity of care. Our findings on the importance of psychological and social support echo research from Australia [18]. PCPs can provide comprehensive psychosocial support through: conducting regular emotional assessments and family coping evaluations; facilitating family meetings; providing anticipatory guidance about disease progression; connecting patients with community resources; and offering ongoing bereavement support [11, 18]. Research demonstrates that PCPs who receive targeted psychosocial training report increased confidence and improved patient outcomes [32].

### Implications for practice

These findings have important implications for healthcare practice in Singapore. Healthcare institutions should develop structured pathways that clearly define PCP roles in palliative care while providing adequate resources and training [33]. Educational curricula should incorporate comprehensive palliative care training, emphasizing communication skills and psychosocial support competencies. When PCPs have access to specialist consultation and clear protocols, they demonstrate greater willingness to engage in EOL care discussions and symptom management [34].

Investment in inter-professional collaboration initiatives that foster mutual understanding between primary and specialist care providers is essential for improving care coordination and reducing role uncertainty. Addressing both systemic and personal factors through educational initiatives focusing on core palliative care competencies, clearer role definitions, and strengthened specialist-PCP collaboration could help bridge the gap between PCPs’ theoretical understanding of their potential roles and practical implementation.

## Limitations

This is the first published study that provides insights into PCPs’ perceptions of their roles in palliative care within Singapore’s public polyclinic setting. However, several limitations should be acknowledged. First, the study was limited to PCPs in SingHealth polyclinics and may not reflect the views of those in the two other health clusters in Singapore. Second, private or home-based care settings, where roles and support structures may differ, could provide different perspectives. Third, the majority of participants were female physicians, which may have influenced findings, as gender differences in attitudes toward psychosocial support and specialist consultation have been observed in healthcare settings [35]. Fourth, the study focused solely on doctors’ perspectives. Future research should incorporate views from other stakeholders, including nurses, allied health professionals, patients, and caregivers, to provide a more comprehensive understanding of primary care’s role in palliative care delivery. Fifth, the participants were relatively young, with ages ranging from 30 to 54 years and years of practice ranging from 4 to 12.5 years. This may have influenced the findings, as more experienced PCPs with longer careers and broader exposure to patients with life-limiting illnesses may hold different perspectives; this limitation might potentially limit the range of views captured. Additionally, including more balanced gender representation and participants from all health clusters and care settings would enhance the transferability of findings to similar healthcare contexts.

## Conclusion

This study explored the perceptions of PCPs in Singapore regarding their roles in palliative care, revealing both a recognized potential for significant contribution and notable reservations. PCPs identified important roles they could play across the palliative care continuum, including symptom management, psychosocial support, advance care planning, and care coordination. However, they also expressed uncertainty stemming from variability in training and clinical exposure, unclear role boundaries, and discomfort with EOL conversations. Systemic and cultural factors, including patient and specialist expectations and the traditional framing of primary care roles, further shaped their views. To better integrate palliative care into primary care, targeted training, clearer role definition, and stronger collaboration with specialist services are essential. Enhancing institutional support and fostering confidence among PCPs may ultimately improve the accessibility, continuity, and quality of care for patients with life-limiting illnesses in the community.

## Supplementary Information

Supplementary Material 1.

## Data Availability

The datasets generated and/or analysed during the current study are not publicly available due to the potential for compromising participant confidentiality, but are available from the corresponding author on reasonable request and with approval from the SingHealth Centralised Institutional Review Board.
