# Supplementary material for: Exploring primary care doctors’ perceptions on their roles in palliative care in Singapore: a qualitative study
Source: BMC Prim Care. 2026 Apr 21;27:221. doi: 10.1186/s12875-026-03330-5 (PMC13231754; doi:10.1186/s12875-026-03330-5)
Supplement: Supplementary file 1 — Supplementary Material 1. [file 12875_2026_3330_MOESM1_ESM.pdf]

Supplementary File 1 – Interview Guide for Primary Care Physicians

Section / Topic	Main Question	Probes / Follow-up Questions
1. Opening Questions	Have you encountered patients who need palliative care?	—
	Can you share your experience in managing them?	—
2. Views and Attitude	How do you identify patients in need of palliative care?	Based on clinical diagnosis? Stage of organ failure? Supportive vs palliative (end-of-life vs terminal stage)
	In your opinion, what is palliative care?	Cancer, chronic illness, life-limiting illness
	Do you think primary care is an appropriate setting to provide palliative care?	Do you think primary care doctors have a role? What is the advantage of introducing palliative care in primary care?
	What do you think about discussing advance care planning with patients who need palliative care?	—
3. Colleagues' Usual Practice (Subjective Norms)	Are you aware of any colleagues in your clinic who are providing palliative care?	What do you think about this? What do your colleagues normally do when they encounter such patients?
	What do you do when you encounter a patient who needs palliative care?	Where do you refer them and why? What is patient's experience with the referral? Any challenges? Are you aware of the outcome of the referral? How important is it for primary care doctors to know what happens to these patients?

4. Potential Barriers (Perceived Behavioural Control)	What are the barriers you face when managing patients requiring palliative care?	—
	What are the reactions from patients & families when you discuss palliative care?	Do you face challenges when discussing palliative care with patients (and families)?
	What drugs are available in the polyclinics for patients needing palliative care?	Pain, shortness of breath, nausea/vomiting, depression/anxiety, etc. What do you normally use to manage these symptoms?
	Is time a constraint to provide palliative care?	—
	Are there any available resources to provide palliative care in your clinic?	Palliative care guideline/protocol, health education materials, medications (opioids), access to specialist clinic, community resources
5. Knowledge and Skills	How confident are you in managing patients who need palliative care in your clinic?	If confident, why? If not, why not?
	What would help increase your confidence in providing palliative care?	Use of guidelines? Practicality? Training attended? Usefulness?
	Should other healthcare workers be trained in palliative care?	Why? Why not?
6. Salience of the Behaviour	Apart from knowledge, what are the important qualities a primary care doctor should have to deliver effective palliative care?	Communication skills, empathy/compassion, teamwork/collaboration, cultural competency, moral/ethics
7. Habit	Do you routinely ask about palliative care needs in patients who may require it?	If yes, why? If not, why not?
